# Migraine-like headaches associated with nickel allergy requiring removal of atrial septal defect closure device

**DOI:** 10.1007/s12055-021-01155-8

**Published:** 2021-03-01

**Authors:** Prasanth Sadasivan Nair, Joseph Swaminadan Jaya, Andrea Comella, Julian Anderson Smith, Richard Harper, Prashant Prakash Joshi

**Affiliations:** 1 Department of Cardiothoracic Surgery, Monash Medical Centre, Level 3, 246 Clayton Road, Clayton, Victoria 3168 Australia; 2 Monash Heart, Monash Medical Centre, Level 2, 246 Clayton Road, Clayton, Victoria 3168 Australia

**Keywords:** Atrial septal defect, Congenital heart defect, Occluder, Nickel allergy

## Abstract

There is a deficit of literature regarding the association between nickel allergy–induced symptoms and implanted devices. This report describes a case of nickel allergy causing debilitating migraine-like symptoms, failing to resolve with medical therapy, requiring surgical removal of the device and repair of the defect.

## Introduction

Nickel is a widely available metal used in a variety of implanted medical devices. Currently, nickel allergy is the most common cause of contact dermatitis in the industrial world, affecting women more commonly. Implants such as atrial septal defect (ASD)/patent foramen ovale (PFO) closure devices have the potential to produce symptoms related to nickel toxicity or hypersensitivity, which can pose diagnostic and management challenges.

## Case report

A 35-year-old female presented with a constellation of symptoms including severe migraine, following percutaneous device closure of an ostium secundum ASD. She had been thoroughly investigated by a cardiologist, a neurologist, and an immunologist and had not responded to medical therapy, including clopidogrel and a course of corticosteroids.

Around two and a half years ago, patient’s large 2.5-cm secundum ASD, with minimal antero-superior rim and small posterior rim, was satisfactorily closed percutaneously with a 27-mm Figulla® Flex 2 Occlutech ASD occluder. Since the implantation, she had consistently complained of headaches, described as waves of pain around her head, rushing to her ears associated with extreme sensitivity to both noise and light. She also described the sensation of chest tightness which occasionally radiated to her back. She was fatigued, confused, and dizzy at times and generally detached psychologically. Clinical examination did not reveal any physical abnormalities.

Prior to the ASD closure, the patient did have a history of migraine and bulimia nervosa. She did not have any other significant medical history. Consideration was given to the possibility that her symptoms may be attributed to an allergic response to the implanted ASD device. She underwent skin allergy testing and was found to be strongly positive for nickel allergy. Serum nickel levels were normal (2 nmol/L, reference value <29 nmol/L). However, this is often the case in patients with nickel allergy, as this is a case of allergy not toxicity.

## Management

After discussion in a multi-disciplinary meeting, it was decided to offer surgical removal of the device. Informed consent was obtained, including the fact that she may not get symptomatic relief after device removal.

**Fig. 1:**
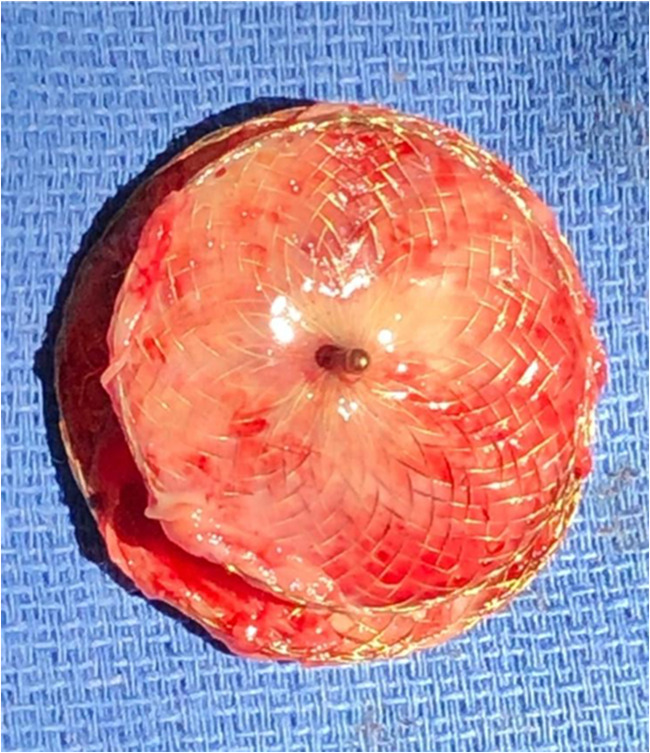
Right atrial surface of the explanted 27-mm Figulla® Flex 2 Occlutech ASD occluder

**Fig. 2:**
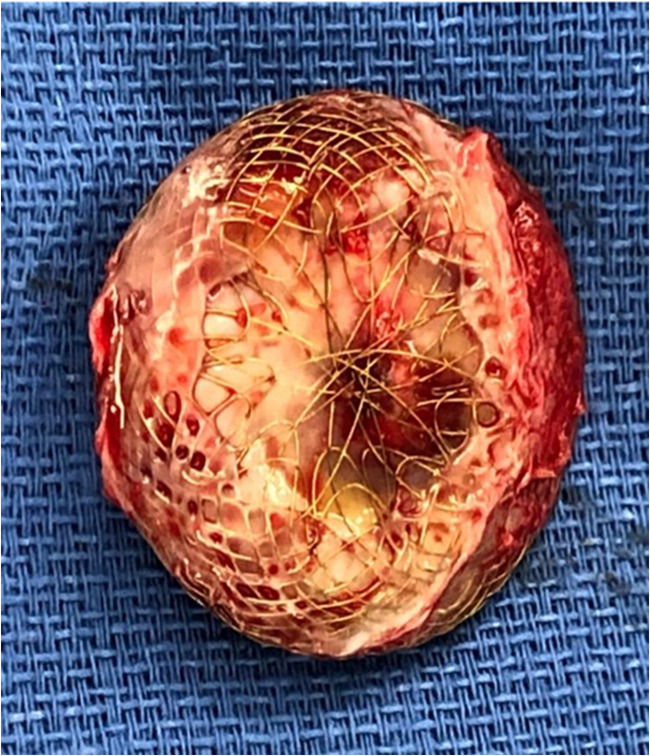
Left atrial surface of the explanted ASD device

**Fig. 3:**
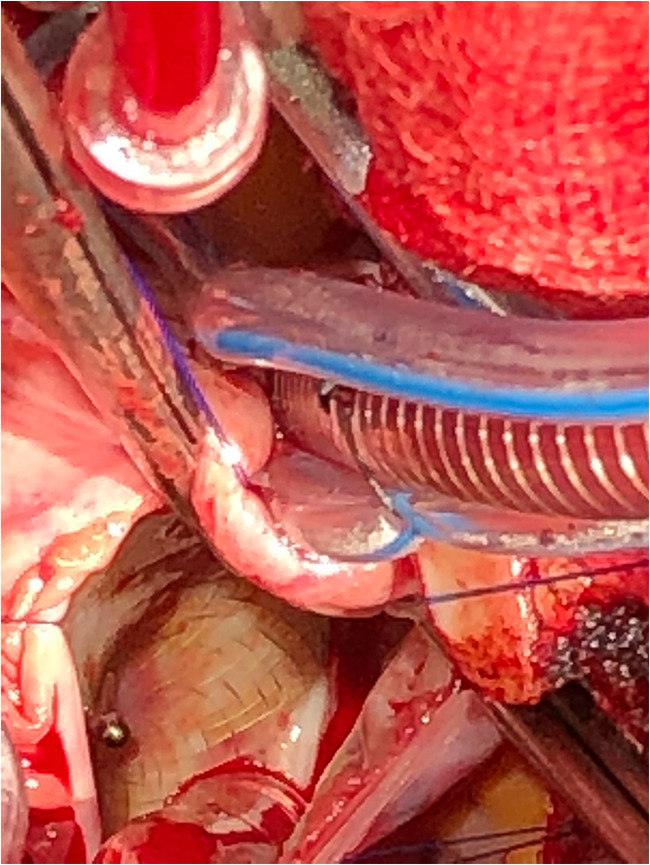
Intraoperative photograph showing the incompletely endothelialised ASD device after the right atriotomy was performed

The operative approach chosen was via a right anterior thoracotomy, respecting the patient’s desire to avoid a midline scar on her chest. A 5-cm skin incision was made in the infra-mammary fold on the right side. Dissection proceeded down to the fourth intercostal space, which was entered, also with division of the costochondral junction of the fifth rib. The pericardium was opened and a large portion excised for subsequent use for patch closure. The patient was heparinised and cardiopulmonary bypass was established by cannulating the ascending aorta and placing cannulae in the superior and inferior vena cavae, which were snared. The ascending aorta was clamped, using a clamp delivered through a separate incision in the chest wall, and the heart was arrested by delivery of cold blood cardioplegia via the aortic root. The right atrium was opened obliquely. The ASD device was incompletely re-endothelialised (Figs. 1 and 2), especially the left atrial surface. The metallic axial portion was also exposed on both surfaces. There was no thrombus in either atria or any adherent to the device (Fig. 3). The device was painstakingly excised, leaving an adequate rim of residual tissue around the ASD. Following removal of the device, the autologous pericardial patch was used to close the defect, using a continuous running 4-0 polypropylene suture. Prior to tying off the suture line, the left side of the heart was thoroughly de-aired, along with resumption of ventilation. The right atriotomy was closed with a continuous running 5-0 polypropylene suture in two layers. The cross clamp was then released, after delivery of terminal warm shot, and weaning off cardiopulmonary bypass was uneventful. Decannulation was done after administering protamine and the thoracotomy wound was closed in layers, over a single drain in the right pleura. Good hemodynamic parameters were achieved on transfer to intensive care with the patient in sinus rhythm. The patient was extubated in intensive care after 4 h. Her in-hospital recovery was complicated by significant neuropathic pain from her thoracotomy wound and subsequent deconditioning. Her pain was controlled with assistance from hospital acute pain services and she was discharged to inpatient rehabilitation 10 days postoperatively. She was discharged home after a brief period of rehabilitation.

## Discussion

Percutaneous closure of secundum ASD is generally a safe and effective procedure. The device used in this case is composed of nitinol, an alloy of 45% titanium and 55% nickel [1]. Although the brand purports that the device is coated with titanium oxide to inhibit nickel release, the amount of nickel eluted from the device is unclear.

There have been several case reports of migraine associated with nickel allergy after device closure [2–5]. These are usually managed conservatively with medical therapy and generally respond well. A quality of life study conducted in Salt Lake City, UT, examined 58 patients who had their ASD devices removed over a period of 10 years. The authors administered questionnaires focused on symptoms, quality of life, and satisfaction, along with a 36-item Short Form Health Survey to measure physical and mental health post-surgery. The paper proposed a management plan with removal of the device for patients with symptomatic PFO or ASD device rejection, linking 3 defined points: the presence of PFO/ASD device, symptoms (preferentially new-onset symptoms that occur relatively soon after device placement), and positive dermatologic evidence of nickel hypersensitivity, either by patch and/or scratch testing [6]. Approximately 7–11% of the general population have been noted to have nickel allergy [7]. The structure of most ASD closure devices is similar, with a nitinol wire mesh shaped into two round discs connected by a short waist.

The purported immunological mechanism underlying nickel allergy is a type IV or cell-mediated hypersensitivity reaction [7]. Direct exposure to nickel through nitinol-containing prosthetic implants, such as ASD or PFO occlusion devices, has been known to cause chest discomfort [8], dyspnea, fever, edema, palpitations, migraine, and pericarditis with effusion. The mechanism is unknown, but there are two proposed pathways. One hypothesis is the induction of a local inflammatory reaction by the device that may result in platelet adhesion that could then embolise to the brain, causing micro-infarcts and migraine headache. However, in our case, despite a recent magnetic resonance imaging (MRI) showing signs of micro-infarcts at the time of explantation, there was no sign of thrombus on the left atrial disc. The second proposed mechanism is that the localised reaction causes release of inflammatory mediators, such as plasma calcitonin gene–related peptide, a protein known to be released from specific cardiac tissue into the left atrium, which then travels to the cerebral circulation and induces migraine headache [9].

Device explantation for nickel hypersensitivity, or other reasons, such as device migration, is not without risks altogether. Dissection to remove the device may inadvertently cause injury to the conduction system, causing heart block, and care must be taken to carefully explant the device leaving adequate rim of septal tissue, especially near the triangle of Koch. The device can also impinge on the mitral/tricuspid apparatus and a thorough examination of the valve apparatus must be carried out to exclude any damage after explantation. Care must also be taken while taking suture bites at the superior rim of the ASD to avoid entrapping the aortic valve leaflets.

There is a statistically significant increase in plasma or urine nickel levels after ASD device implantation, up to a fivefold increase in some cases. These high levels may occur before the device re-endothelialises or a calcium phosphate layer forms over the device [10] and the nickel levels fall toward baseline in 6–12 months after insertion. The reason for inadequate endothelialisation, late in the postoperative period, in some patients warrants further study.

## Follow-up

At a 3-month follow-up appointment, the patient’s headaches had completely resolved. Her associated visual disturbance had also improved.

## Conclusions

Nitinol is widely used in medical products because of its good radio-opacity and shape memory properties. Speculation remains about the potential release and hypersensitivity reactions to nickel. Nickel hypersensitivity associated with ASD occluders is purported to be caused by immune mechanisms.

After ASD closure using an occluder, an increased incidence of migraine headache during the post-procedural course was shown to be correlated with nickel hypersensitivity and large device size. Pharmacologic suppression of platelet aggregation on the implanted device may prevent embolisation. Nickel hypersensitivity–related symptoms might persist for several months, but usually respond to medical therapy, including antihistamines, steroids, or the addition of clopidogrel for 3 months.

In rare cases, in which medical management fails, surgical removal of the device may be considered. When performing intra-cardiac implantation with nickel-based devices, a patch test for nickel hypersensitivity does not appear to be applied systematically, because of its lack of sensitivity and specificity. Majority of the patients who have ASD/PFO devices implanted and are found to have documented nickel allergies do not experience any symptoms. Hence, presence of documented nickel allergy cannot be seen as a contraindication to ASD/PFO device implantation.

We advocate early removal of the device in patients with symptoms of nickel hypersensitivity that remains persistent after 12 months, and a clear explanation of possible symptoms of nickel allergy should be made to the patients before device implantation.
